# Role of Chest X-Ray in Coronavirus Disease and Correlation of Radiological Features with Clinical Outcomes in Indian Patients

**DOI:** 10.1155/2021/6326947

**Published:** 2021-10-06

**Authors:** Satyanand Sathi, Richa Tiwari, Savita Verma, Anil Kumar Garg, Virendra Singh Saini, Manoj Kumar Singh, Amit Mittal, Devinder Vohra

**Affiliations:** ^1^Department of Medicine, S.M.M.H. Government Medical College Saharanpur, Saharanpur, Uttar Pradesh, India; ^2^Department of Radiology, S.M.M.H. Government Medical College Saharanpur, Saharanpur, Uttar Pradesh, India; ^3^Department of Obstetrics and Gynaecology, S.M.M.H. Government Medical College Saharanpur, Saharanpur, Uttar Pradesh, India; ^4^Department of Community Medicine, S.M.M.H. Government Medical College Saharanpur, Saharanpur, Uttar Pradesh, India

## Abstract

Recent literature has reported that radiological features of coronavirus disease (COVID-19) patients are influenced by computed tomography. This study aimed to assess the characteristic chest X-ray features of COVID-19 and correlate them with clinical outcomes of patients. This retrospective study included 120 COVID-19 patients. Baseline chest X-rays and serial chest X-rays were reviewed. A severity index in the form of maximum radiological assessment of lung edema (RALE) score was calculated for each lung, and scores of both the lungs were summed to obtain a final score. The mean ± standard deviation (SD) and frequency (%) were determined, and an unpaired *t* test, Spearman's rank correlation coefficient, and logistic regression analyses were performed for statistical analyses. Among 120 COVID-19 patients, 74 (61.67%) and 46 (38.33%) were males and females, respectively; 64 patients (53.33%) had ground-glass opacities (GGO), 55 (45.83%) had consolidation, and 38 (31.67%) had reticular-nodular opacities, with lower zone distribution (50%) and peripheral distribution (41.67%). Baseline chest X-ray showed a sensitivity of 63.3% in diagnosing typical findings of SARS-CoV-2 pneumonia. The maximum RALE score was 2.13 ± 1.9 in hospitalized patients and 0.57 ± 0.77 in discharged patients (*p* value <0.0001). Spearman's rank correlation coefficient between maximum RALE score and clinical outcome parameters was as follows: age, 0.721 (*p* value <0.00001); >10 days of hospital stay, 0.5478 (*p* value <0.05); ≤10 days of hospital stay, 0.5384 (*p* value <0.0001); discharged patients, 0.5433 (*p* value <0.0001); and death, 0.6182 (*p* value = 0.0568). The logistic regression analysis revealed that maximum RALE scores (0.0932 [0.024–0.367]), (10.730 [2.727–42.206]), (1.258 [0.990–1.598]), and (0.794 [0.625–1.009]) predicted discharge, death, >10 days of hospital stay, and ≤10 days of hospital stay, respectively. The study findings suggested that the RALE score can quantify the extent of COVID-19 and can predict the prognosis of patients.

## 1. Introduction

The World Health Organization coined the official term coronavirus disease (COVID-19) on February 11, 2020, and declared it to be a pandemic on March 12, 2020 [1, 2]. The real-time reverse transcription polymerase reaction (RT-PCR) of viral nucleic acid has limited reliability due to a high number of false negative results; a correlation of clinical features with the findings on a chest radiograph (CXR) would be more definite for a confirmatory diagnosis of COVID-19. On CXR, changes may be ground-glass opacities (GGO), horizontal linear opacities, or consolidation [3]. The CXR findings in COVID-19 patients are usually most evident about 10–12 days after onset of symptoms [4]. Andrea Borghesi and Roberto Maroldi suggested the two-step Brixia scoring system for COVID-19 [5]. In the first step, both the lungs were divided into 6 zones; in the second step, a score (from 0 to 3) was given to each zone on the basis of detection of lung abnormalities; this gave an overall CXR score ranging from 0 to 18 [5]. Warren et al. suggested the use of the radiographic assessment of lung edema (RALE) score for the assessment of density of alveolar opacities, and the scoring system was adopted by Wong et al., Yasin and Gouda, Kaleemi et al., and Warren et al. in their studies [4, 6–8]. In RALE scoring, each CXR was given a score between 0 and 8, ranging from the absence of any involvement (score 0) to complete lung involvement on both sides (score 8).

Excessive transmission of COVID-19 has shown to have an adverse impact on the economy of developing countries with inadequate healthcare infrastructure. The CXR may be considered as an inexpensive first-line investigative modality for detection of abnormalities in lung parenchyma. We hypothesized that systematic quantitative details on the CXR could be helpful to delineate COVID-19 severity and modify the treatment plan accordingly. The aim of our study was to understand the characteristics of CXR features of COVID-19 in a structured way and to correlate radiological features with the patient's clinical outcome (length of hospital stay, discharge, and death).

## 2. Materials and Methods

The CXRs of 120 confirmed COVID-19 patients, performed either in an isolation ward or in an intensive care unit (ICU) of our institute from July 15 to July 31, 2020, were reviewed retrospectively. The duration of the study was 17 days. The inclusion criteria were age ≥18 years and a diagnosis of COVID-19 confirmed through positive naso- or oropharyngeal swabs for SARS-CoV-2 RNA-PCR. The following exclusion criteria were applied: age <18 years, pregnancy, and patients who left against medical advice. The final clinical outcome was demonstrated as length of hospital stay, discharge, and death. The study was approved by the institutional ethics committee of Shaikh-Ul-Hind Maulana Mahmood Hasan (SMMH) Government Medical College, Saharanpur, UP, India.

### 2.1. Image Acquisition and Analysis

**Figure 1:**
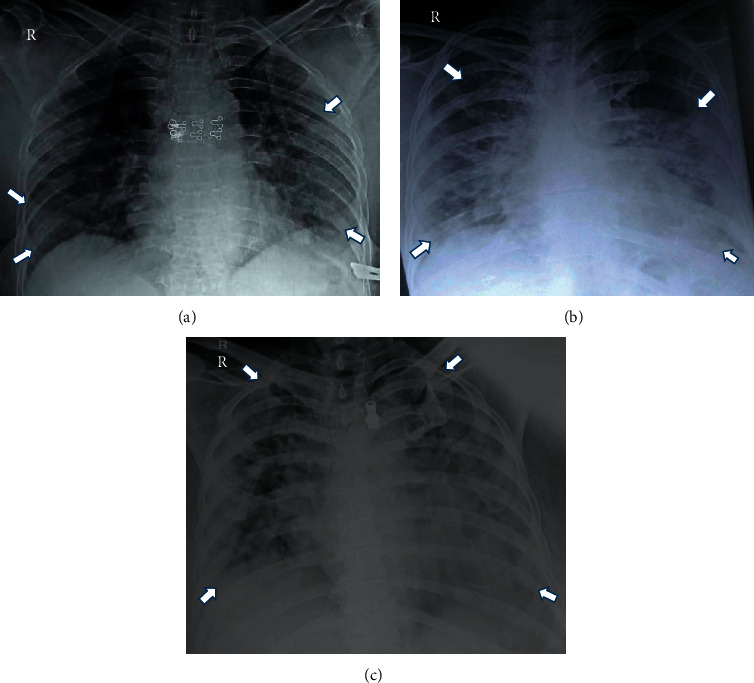
Evolution of chest X-ray findings in a COVID-19 patient with an X-ray scoring system. (a) Day 3: RALE score: 2 + 3 = 5. (b) Day 7: RALE score: 4 + 3 = 7. (c) Day 9: RALE score: 4 + 4 = 8 patient died on day 10.

**Figure 2:**
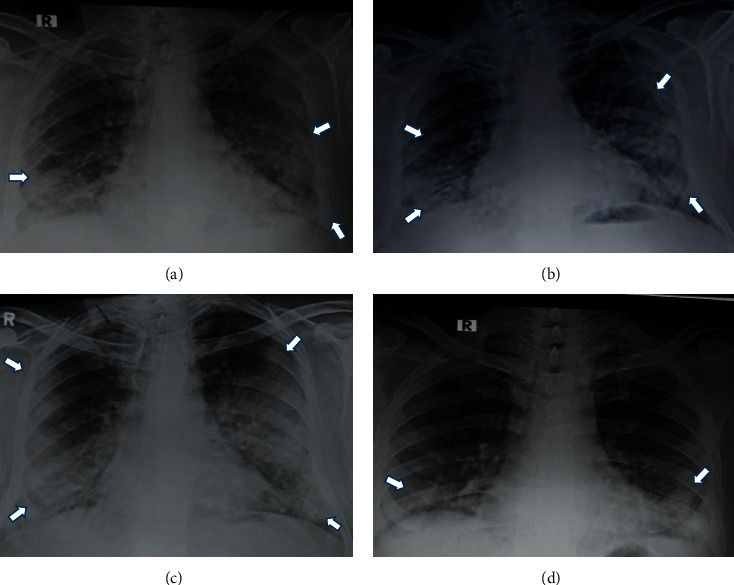
Evolution of X-ray findings in a COVID-19 patient. (a) Day 3: RALE score: 3. (b) Day 6: RALE score: 5. (c) Day 10: RALE score: 6. (d) Day 14: RALE score: 2 patient was discharged on day 21.

All CXRs were performed as digital radiographs following hospital protocols with the same portable X-ray unit (Medilux systems portable 100 ma X-ray machine) in an isolation ward or in an ICU. CXRs were acquired in anteroposterior (AP) projection for bed-ridden patients and in the posteroanterior (PA) projection for the rest of the patients; follow-up CXRs were performed similarly. One senior radiologist reviewed each CXR in order to define the radiological aspects of COVID-19. According to the Fleischer Society glossary of terms, radiological features were identified as ground-glass opacities (GGO), consolidation, reticular-nodular opacities, and pulmonary nodules [9]. Moreover, specific patterns of lung involvement were characterized as perihilar predominance or peripheral predominance (demarcation between two was defined as halfway between the hilum and the lateral edge of the lung), unilateral or bilateral lung involvement, and lower zone, upper zone, or no zonal preference. Other associated pulmonary pathologies such as pleural effusion, cardiomegaly, and pneumothorax were also recorded. The extent of lung involvement was calculated as severity score by applying Radiographic Assessment of Lung Edema (RALE) score which was suggested by Warren et al. [8]. Depending on consolidation or ground-glass opacity (GGO), each CXR was given a score between 0 and 8, ranging from the absence of any involvement (score 0) to complete lung involvement on both sides (score 8) [4, 6, 7]: score 0; no involvement of lung parenchyma, score 1; <25% involvement of lung parenchyma, score 2; 25–50% involvement of lung parenchyma, score 3; 50–75% involvement of lung parenchyma, and score 4; >75% involvement of lung parenchyma. The RALE score for each patient was calculated from that CXR which showed maximum involvement of lung parenchyma among all CXRs performed during the hospital stay of the patient, and the final severity score (maximum RALE score) was calculated by summing of maximum scores of each lung. RALE score was also calculated at the time of discharge for all patients. Examples are shown in Figures 1 and 2. Use of maximum RALE score on CXRs reduces heterogeneity as it provides a set standard of the maximum involvement of lung parenchyma, and we can compare clinical outcome of patients against it. This scoring system was utilized to assess its prognostic value in terms of clinical outcome of patients. Previously, this scoring system was used by Wong et al. and Kaleemi et al. [4, 7]. On the basis of maximum RALE score, severity of lung involvement was classified as mild (maximum RALE score of 1-2), moderate (maximum RALE score of 3-4), severe (maximum RALE score of 5-6), and very severe (maximum RALE score of 7-8). A similar type of severity score (1–6) was used by Wong et al. for baseline CXR, and they did not mention score 7-8 as none of their patients had a severity score of >6 on baseline CXR [4].

### 2.2. Statistical Analysis

Data were analysed by Statistical Package for Social Sciences (SPSS) software (version 18.0 for Windows). Values are presented as mean ± standard deviation. In case a normal distribution for categorical variables could not be established, frequency (%) was presented instead. An unpaired *t* test was applied to discern possible differences between the means of RALE score estimated in the two groups: hospitalized patients and discharged patients. Spearman's rank correlation coefficient was calculated to find out the correlation between RALE score and clinical outcome parameters. Logistic regression analysis was performed with the variables being maximum RALE score, recovery, death, and length of hospital stay. *p* value ≤ 0.05 was considered as statistically significant.

## 3. Results

The clinical characteristics of 120 patients on presentation are shown in Table 1. There were 74 males (61.67%) and 46 females (38.33%) with a mean age of 50 years (range 18 to 85 years).

**Figure 3:**
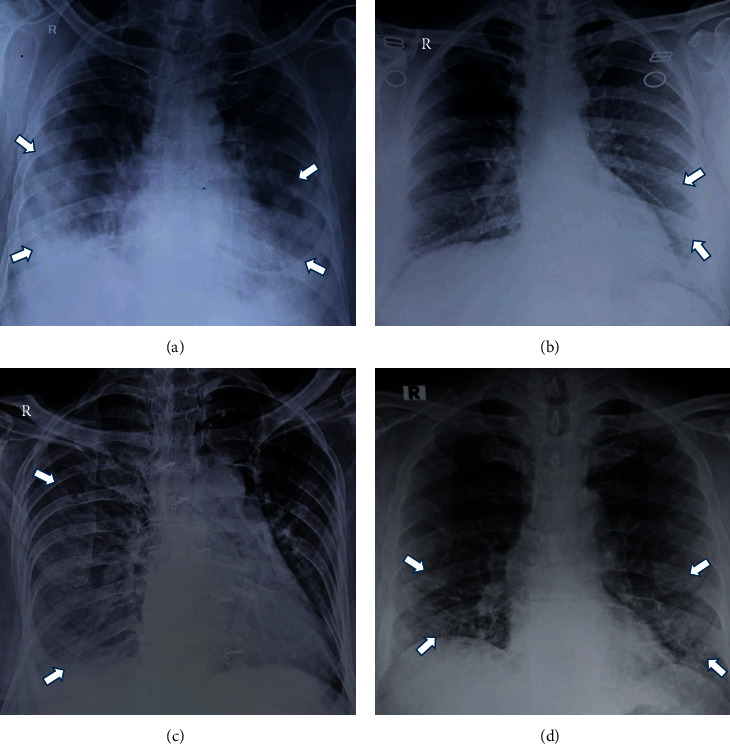
The spectrum of X-ray findings in COVID-19 patients. (a) Consolidation. (b) Ground-glass opacities (subtle GGO in the left lower lung zone). (c) Pleural effusion (veil-like opacity in the right hemithorax). (d) Reticulonodular pattern.

**Figure 4:**
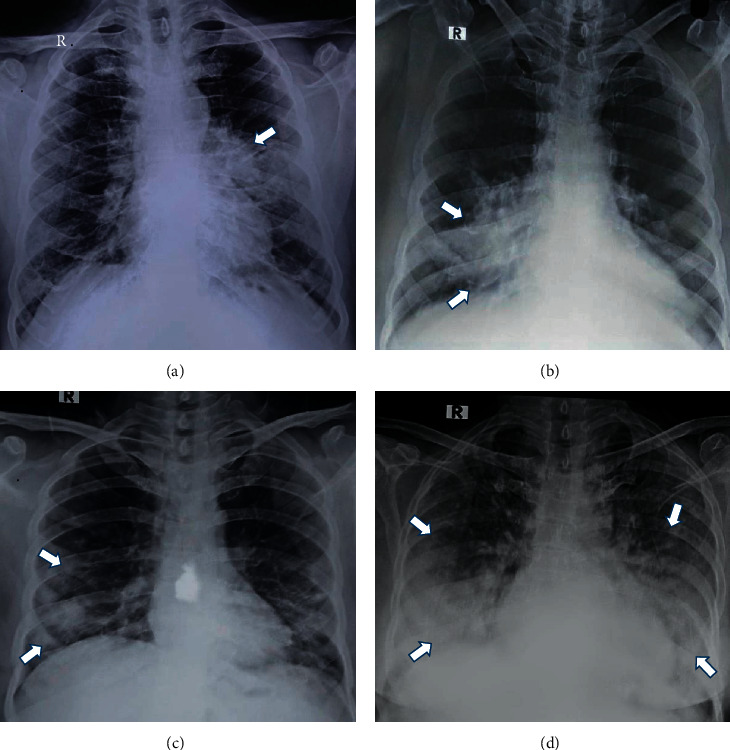
The various patterns of consolidation. (a) Central-perihilar. (b) Central-infrahilar. (c) Peripheral. (d) Diffuse and basal.

The most common comorbid conditions were diabetes (15%) and hypertension (10%). The mean of maximum RALE score was 2.13 ± 1.9 and 0.57 ± 0.77 for 120 hospitalized patients and 110 discharged (recovered) patients, respectively. The difference of RALE score between these two groups was statistically significant (*p* < 0.0001) (Table 1). All patients underwent baseline CXRs upon presentation. Eighty-nine of 120 patients demonstrated abnormalities on CXRs at some point during their hospital stay (Table 2). Thirty-one patients (25.83%) had normal CXRs throughout their hospital stay. The spectrum of CXR findings and various patterns of consolidation are shown in Figures 3 and 4.

**Figure 5:**
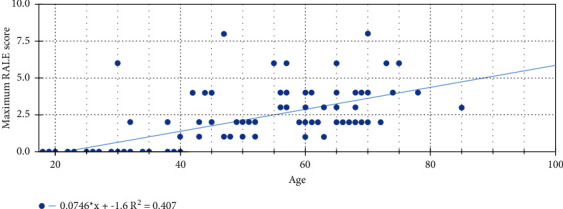
Correlation between age and maximum RALE score, representing the severity score (mild 1-2, moderate 3-4, severe 5-6, and very severe 7-8).

**Figure 6:**
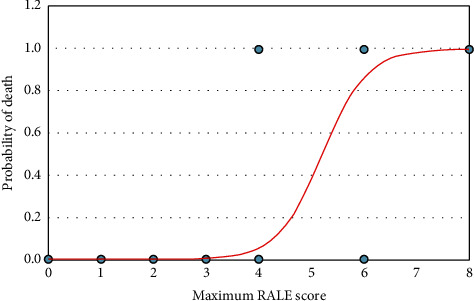
Graph of a logistic regression curve showing probability of death versus maximum RALE score.

CXRs showed mild involvement in 54 patients (45%) who had a maximum RALE score of 1-2 during hospital stay. Moderate involvement was found in 25 patients (20.83%) who had a maximum RALE score of 3-4 during their hospital stay. Severe involvement was found in 7 patients (5.83%) who had a maximum RALE score of 5-6 during their hospital stay. Very severe involvement was found in 3 patients (2.5%) who had a maximum RALE score of 7-8 during their hospital stay. The highest possible CXR RALE score (severity score) was recorded as 8 in 3 patients. Out of a total of 10 deaths, 9 were observed among the patients who had a maximum RALE score of 6 to 8 during their hospital stay. Out of forty-four patients who had normal baseline CXRs, 13 patients (10.83%) developed abnormalities on follow-up CXRs; out of these 13 patients, no one died during their hospital stay. Peak severity of RALE score was reached at 9 to 12 days of onset of symptoms. The length of hospital stay was from minimum 1 day to maximum 21 days. Nineteen patients had a hospital stay of >10 days; out of these 19 patients, 18 recovered and only 1 patient died. The correlation of maximum RALE scores with the clinical outcome and age of the patients is shown in Table 3 and in Figure 5. Results of logistic regression analysis for maximum RALE score are shown in Table 4. On logistic regression analysis, the maximum RALE score (0.0932 [0.024–0.367]) was found to be a predictor for discharge (recovery) of patients. The maximum RALE score 6–8 (10.730 [2.727–42.206]) was found to be a predictor of death of the patients (Figure 6). The maximum RALE scores of 1.258 (0.990–1.598) and 0.794 (0.625–1.009) were predictors of a hospital stay of >10 days and ≤10 days, respectively.

## 4. Discussion

In the course of the ongoing pandemic, the CXR can help in rapid identification of patients with suspected COVID-19. The gold standard diagnostic test for SARS-CoV-2 is considered to be RT-PCR of viral nucleic acid. However, this test has some shortcomings due to delay in reporting and a rising number of false negative results. When there is strong clinical suspicion of COVID-19 and a report of RT-PCR for SARS-CoV-2 is waited for diagnosis, a rapid radiological evaluation is mandatory to initiate early optimal treatment. The CXR should be recommended as the first-line imaging modality, while a computed tomography (CT) scan remains the imaging modality of choice in particular cases [10, 11]. A CT scan has low specificity and very high sensitivity (approximately 97-98%) in diagnosing typical findings of SARS-CoV-2 pneumonia [12, 13]. In our study patients, baseline CXR had a sensitivity of 63.3% whereas the most recent literature showed a variability between 69 and 90 percent [4, 12]. In our institute, CXR is usually performed before CT examination. A CT scan is performed only in specific conditions whenever there is clinicoradiological mismatch such as negative CXR for symptomatic infective lung disease, patients having severe respiratory failure, and suspicion of pulmonary embolism or malignancy. A portable X-ray machine is of value because of easy availability, faster results, less radiation exposure, easy disinfection procedure, and minimum risk of cross infection. Community-acquired pneumonias on CXRs are predominantly unilateral and involve only one part of the lung; there are no ground-glass or linear opacities, and they are typically associated with consolidation on CXRs [3]. In our study, CXRs showed patchy or diffuse opacities and consolidation with bilateral, basal, and peripheral predominance in a majority of the patients (Figures 3 and 4). Among opacities, the most commonly observed were ground-glass opacities followed by consolidation and reticular-nodular opacities (Table 2). These findings were consistent with the main radiological features which were demonstrated in previous studies in COVID-19 patients [3, 14]. The CXRs of our study patients were performed between three to fourteen days after the onset of symptoms. Advanced lung involvement was found between nine to twelve days of onset of symptoms, and it was consistent with the study performed by Wong et al. [4]. RALE score was used to assess the extent of lung involvement and to estimate a prognostic score in the study patients [9]. Our study showed a statistically significant correlation between maximum RALE score and clinical outcome of the patients (Table 3). The maximum RALE score was positively and significantly correlated with age (*r* = 0.721) (Figure 5), and it was consistent with the studies conducted by Kaleemi et al. and Salmeron et al. [7, 15]. The maximum RALE score was positively correlated with the length of hospital stay and the death of patients, and it was consistent with the study conducted by Kaleemi et al. [7].

On logistic regression analysis, the maximum RALE score (0.0932 [0.024–0.367]) was found to be a predictor for discharge (recovery) of patients. In our study, the maximum RALE score predicted the death of patients (10.730[2.727–42.206]) (Figure 6), and it was consistent with the study conducted by Kaleemi et al. [7]. The CXR severity score (Brixia score, 0 to 18) designed by Borghesi et al. predicted mortality in the same way [5]. The maximum RALE score was found to be a predictor of length of hospital stay; Kaleemi et al. observed a longer length of hospital stay among intubated patients [7]. Our study showed that the maximum RALE scores (1.258 [0.990–1.598] and 0.794 [0.625–1.009]) were predictors for a hospital stay of >10 days and ≤10 days, respectively.

Thus, data from our study demonstrate that RALE score can be considered as a valid systematized prognostic score. At times, lung involvement in COVID-19 occurs rapidly; hence, daily or alternate-day clinical monitoring of patients, especially of those in the ICU, by a CXR is recommended. However, daily CXRs are not recommended by the Fleischner Society in stable intubated patients [16, 17]. We believe that clinical and laboratory parameters such as serum D-dimer, serum ferritin, C-reactive protein, and interleukin-6 should always be correlated with radiological imaging to monitor the course of disease. Evidently, there are different radiological protocols in hospitals of different countries for the management of COVID-19 patients, but the safety of health professionals should be prioritised while utilising the diagnostic modalities [16].

Our study has many limitations. Firstly, it is a retrospective study and assessment of sensitivity and specificity of CXRs has been limited as there is an absence of a non-COVID-19 control group for the estimation of prognostic scores. Secondly, the patient's comorbid conditions are not taken into consideration while comparing and correlating the RALE scores with parameters of clinical outcome. Thirdly, there is a difference in time period between the onset of symptoms and performance of CXRs. These variables should be taken into consideration in future studies, and the variability of RALE score should be evaluated in follow-up CXRs.

## 5. Conclusions

The characteristics of COVID-19 included bilateral, basal, and peripheral predominance on CXR in our study patients. RALE score can quantify the extent of COVID-19 and can predict the prognosis of patients. The baseline CXR showed a sensitivity of 63.3% in study patients. On the basis of the results of our study, we can stratify SARS-CoV-2 pneumonia into mild, moderate, and severe and can initiate optimal treatment accordingly.

## Figures and Tables

**Table 1:** Demographic characteristics and RALE score of hospitalized and discharged patients.

	*N*	%	Mean ± S.D.	*p* value
*Age (years)*				
18–20	4	3.33	19.25 ± 0.95	
20–30	18	15	27.66 ± 2.54	
30–40	18	15	35.72 ± 3.54	
40–50	21	17.50	47.29 ± 2.88	
50–60	20	16.67	56.7 ± 3.54	
60–70	33	27.50	66.21 ± 3.02	
70–80	5	4.17	74.4 ± 2.30	
80–90	1	0.83		
Male	74	61.67		
Female	46	38.33		
Diabetes mellitus	18	15		
Hypertension	12	10		

*Maximum RALE score*				
Hospitalized patients	120	100	2.13 ± 1.9	<0.0001
Discharged patients	110	91.66	0.57 ± 0.77	

**Table 2:** Radiographic features of 120 COVID-19 patients.

Radiological findings	*N*	%
Abnormal baseline chest X-ray	76	63.3
Normal baseline chest X-ray	44	36.67
Appearance of abnormality in follow-up X-ray	13	10.83
Ground-glass opacities	64	53.33
Consolidation	55	45.83
Reticular-nodular opacities	38	31.67

*Maximum RALE score during hospital stay*		
1-2 (mild)	54	45
3-4 (moderate)	25	20.83
5-6 (severe)	7	5.83
7-8 (very severe)	3	2.5

*Pattern of opacity*		
Peripheral	50	41.67
Perihilar	24	20
Basal	60	50
Diffuse	25	20.83
Unilateral involvement	19	15.83
Bilateral involvement	70	58.33
Pleural effusion	18	15
Cardiomegaly	36	30

**Table 3:** The correlations of maximum RALE score with clinical outcome parameters and age of the patients.

Clinical outcome parameters	Maximum RALE score	
	Spearman's rank correlation coefficient	*p* value
Discharge (recovery)	0.5433	<0.0001
Death	0.6182	0.0568

*Length of hospital stay*		
>10 days	0.5478	<0.05
≤10 days	0.5384	<0.0001
Age	0.721	<0.00001

**Table 4:** Logistic regression analysis for maximum RALE score results.

Variable	Maximum RALE score	
	Odds ratio (95% CI)	*p* value
Discharge (recovery)	0.0932 (0.024–0.367)	0.0007
Death	10.730 (2.727–42.206)	0.0007

*Length of hospital stay*		
>10 days	1.258 (0.990–1.598)	0.059
≤10 days	0.794 (0.625–1.009)	0.059

## Data Availability

The datasets used and/or analyzed during the current study are available from the corresponding author upon reasonable request.
